# Impact of daily vitamin D_3_ supplementation on the risk of vitamin D deficiency with the interaction of rs2282679 in vitamin D binding protein gene (GC) among overweight and obese children and adolescents: A one-year randomized controlled trial

**DOI:** 10.3389/fnut.2022.1061496

**Published:** 2022-12-12

**Authors:** Golaleh Asghari, Emad Yuzbashian, Ali Nikparast, Leila Najd Hassan Bonab, Maryam Mahdavi, Maryam S. Daneshpour, Farhad Hosseinpanah, Parvin Mirmiran

**Affiliations:** ^1^Department of Clinical Nutrition and Dietetics, Faculty of Nutrition Sciences and Food Technology, Shahid Beheshti University of Medical Sciences, Tehran, Iran; ^2^Nutrition and Endocrine Research Center, Research Institute for Endocrine Sciences, Shahid Beheshti University of Medical Sciences, Tehran, Iran; ^3^Department of Agricultural, Food and Nutritional Sciences, University of Alberta, Edmonton, AB, Canada; ^4^Cellular and Molecular Endocrine Research Center, Research Institute for Endocrine Sciences, Shahid Beheshti University of Medical Sciences, Tehran, Iran; ^5^Obesity Research Center, Research Institute for Endocrine Sciences, Shahid Beheshti University of Medical Sciences, Tehran, Iran

**Keywords:** vitamin D, polymorphism, rs2282679, deficiency, supplement, children

## Abstract

**Background:**

The rs2282679 polymorphism in the vitamin D binding protein (DBP) gene may influence the response to vitamin D supplementation. Therefore, we examine the effect of 1-year vitamin D supplementation on vitamin D deficiency (VDD) with the interaction of rs2282679 polymorphism in overweight and obese children and adolescents.

**Materials and methods:**

The participants (*n* = 300) were part of a randomized controlled trial who received a daily supplement of either 1,000 or 2,000 IU or four supplements of 1,000 IU weekly (equal to 600 IU daily) of vitamin D_3_ for 12 months. Genotyping was performed using amplification refractory mutation system polymerase chain reaction (ARMS-PCR).

**Results:**

The mean of 25(OH)D values at baseline for participants with the TT, TG, and GG genotypes were 15.4, 14.4, and 10.8 ng/mL, respectively, and were not different between the three genotype groups (*P* = 0.062). A significant reduction in VDD was observed after vitamin D supplementation with dosages of 1,000 or 2,000 IU compared to 600 IU. No significant association of genotypes with risk of VDD was observed in each intervention group after vitamin D supplementation, except, that individuals with TG genotype showed a higher risk of VDD compared to those with TT genotype in the 2,000 IU group after 6 months of supplementation [odds ratio (95% CI): 6.94; 1.30–37.02]. We observed no interaction between time duration, three genotypes, and dosages with serum 25(OH)D, calcium, phosphorus, alkaline phosphatase, and parathyroid hormone levels.

**Conclusion:**

Response to vitamin D supplementation by three doses of 600, 1,000, and 2,000 IU could not be affected by rs2282679 polymorphism during 12 months in overweight and obese children and adolescents.

## Introduction

It is imperative to maintain an adequate level of vitamin D, as assessed by the 25-hydroxyvitamin D [25(OH)D] concentration, since vitamin D deficiency (VDD) has been linked to several medical outcomes, including osteoporosis, cardiovascular heart disease, type 1 diabetes, autoimmune diseases, and some cancers (1). There is alarming evidence that VDD is prevalent in more than one billion people worldwide, particularly children and adolescents (2). A meta-analysis of 24,600 children and adolescents has reported that those with obesity have a 41% higher risk of VDD compared with their normal-weight peers (3). Furthermore, it has been demonstrated that supplementation with vitamin D has a lower response in obese children than non-obese children when given the same dose of vitamin D (4). In addition to obesity, individual responses to vitamin D supplementation may vary based on baseline values, sex, age, and genetic factors, suggesting that ensuring adequate levels may require a tailored approach (5, 6).

When it comes to genetic diversity, it means that different genetic makeup can affect the vitamin D pathway chain, including metabolism, transportation, and signaling (6). Vitamin D binding protein (DBP), which is encoded by the group-specific component (GC) gene, is involved in the transportation of about 85–90% of the circulating vitamin D pool, specifically 25(OH)D (7). Interestingly, Wang et al. have shown that variants in the GC gene including, rs3755967, rs17467825, rs1155563, rs2298850, rs7041, and rs2282679 may determine vitamin D status through the different affinity of DBP for vitamin D (8). Therefore, the identification of GC variants (i.e., rs7041 and rs2282679) and their correlation with DBP concentration has been demonstrated by genome-wide association studies (GWAS) (8, 9). A recent systematic review was conducted to identify published single nucleotide polymorphisms (SNPs), which are associated with the vitamin D pathway. This review has indicated that the highest confirmation rate, association with vitamin D status, was found for the rs2282679 polymorphism in the GC gene (confirmed by 23 out of 30 studies). In the aforementioned SNP, the major allele was the beneficiary one in all of the studies (6).

Several observational studies have indicated that the rs2282679 polymorphism is associated with a lower level of 25(OH)D among children and adolescents (10, 11). Nissen et al. have shown that children and adolescents who were minor allele carriers for rs2282679 polymorphism had lower 25(OH)D concentration compared with peers (10); a finding which has been confirmed among Iranian adolescents (11). In addition to observational studies, several intervention studies have also determined the influence of rs2282679 polymorphism on 25(OH)D response to vitamin D supplementation (12–15). Muindi et al. found that rs2282679 in colorectal cancer patients receiving 2,000 IU daily oral D3 supplementation during 6 months was associated with an increased risk of vitamin D3 insufficiency and suboptimal increase in 25(OH)D3 levels (12).

The results of our previous study showed that doses of 1,000 and 2,000 IU of vitamin D increased 25(OH)D concentrations in comparison to 600 IU/day, while no changes were observed in serum calcium, phosphorus, or alkaline phosphatase levels among overweight and obese school-children (16). To the best of our knowledge, there has not been a study examining the influence of rs2282679 polymorphism on overweight and obese school children’s response to vitamin D supplementation. Therefore, we aimed to investigate the interaction between the duration of vitamin D supplementation, 1,000 and 2,000 IU vitamin D supplements, and rs2282679 genotypes with serum 25(OH)D, calcium, phosphorus, alkaline phosphatase, and parathyroid hormone levels in overweight and obese children and adolescents during 1-year supplementation.

## Materials and methods

### Study subjects

The present study is a *post hoc* analysis of a randomized controlled trial of vitamin D supplementation in overweight and obese children and adolescents (IRCT clinical trial registry number: IRCT20180805040703N1) (16).

Children and adolescents aged 6–13 years old were recruited from primary schools in three different districts of Tehran, Iran (35.7°N). The participants and their parents were invited to the Research Institute for Endocrine Sciences (RIES), Shahid Beheshti University of Medical Sciences (SBMU), to be asked about the data required for the current study. After considering the exclusion criteria (lack of interest, having serious illnesses, and taking vitamin D supplements) and meeting the medical history and examinations, 378 out of 525 individuals were ultimately allocated by randomization. Among 378 individuals, 300 individuals underwent genotyping. The study was approved by the institutional ethics committee of the RIES, affiliated with the SBMU in Tehran (NO: IR.SBMU.ENDOCRINE.REC.1395.326). This study was conducted according to the Declaration of Helsinki’s ethical principles. Parents or guardians obtained written informed consent, in addition to which all children provided assent to participate.

### Randomization

Participants were designated by a computer-generated block randomization program to receive a daily supplement of either 1,000 IU (25 μg) or 2,000 IU (50 μg) vitamin D3 or four supplements of 1,000 IU (25 μg) D3 weekly [equal to 600 IU (15 μg) daily] for 12 months. They were stratified according to their gender, weight status (overweight or obese), and puberty status (pre-pubertal or pubertal). Both doses of vitamin D3 supplements were provided by the Zahravi Pharmacy Co. (Tabriz, Iran). To reevaluate the vitamin D3 content, an independent laboratory (Tajzieh Kimia Novin Azma, Iran) contributed and reported 25.1 μg/for 1,000 IU and 51.1 μg/cps for 2,000 IU. The outcome assessors and data analysts were blinded, but the participants and intervention providers, who were also data collectors, were not blinded to group assignment in the current RCT.

### Covariates

At baseline, guardians (predominantly mothers) were asked sociodemographic and medical history questions. Weight and body composition (percent of body fat and soft lean mass) were assessed by a bioelectric impedance analyzer (BIA; Model, GAIA 359 Plus, Co. Cosmed, Italy), and height was measured to the nearest 0.5 cm in a standing position, without shoes, using a measuring tape while the shoulders were in a normal position. Body mass index (BMI) was calculated as weight (kg) divided by height (m) squared (kg/m^2^). In addition, the World Health Organization child growth standard was used to define 6–19 years old children as overweight (BMI between 1 and 2SD standard curve) and obese (BMI > 2SD standard curve) (17). Tanner stages were examined by a well-trained physician. Information on sun exposure was measured by a questionnaire. Dietary intake was measured by a semi quantitative food frequency questionnaire (FFQ) (18) and physical activity was assessed by the modifiable activity questionnaire (MAQ) (19) during an interview. After fasting for 12–14 h, a blood sample was obtained in order to perform further analysis. The participants consuming ≥80% of pills were considered in the final analysis. All measurements were also evaluated after 6 and 12 months.

### Clinical measurements

Calcium and phosphorus were measured using a photometric method by arsenazo III (20) and UV photometric methods, respectively. Alkaline phosphatase was measured using the kinetic photometric method standardized by Deutsche Gesellschaft Fur Klinische Chemie (DGKC). Calcium, phosphorus, and alkaline phosphatase were measured by Pictus 700 Clinical Chemistry Analyzer, Diatron MI PIc (Budapest, Hungry), and Parsazmoon kits (Tehran-Iran). Intra- and inter-assay coefficients of variation (CVs) were <2.5 for calcium, <3.5 for phosphorous and <3.0 for alkaline phosphatase. 25(OH) D concentrations were determined by the electrochemiluminescence immunoassay (ECLIA) method using the Roche/Hitachi Cobas *e*-411 analyzer (Roche Diagnostics, GmbH, Mannheim, Germany). Intra- and inter-assay CVs were <7.5 for 25(OH)D concentrations.

### Genotyping

DNA samples were extracted from white blood cells by a standard Proteinase K, salting-out method. Spectrophotometry and electrophoresis were performed to evaluate the accuracy and quality of extracted DNA samples (21). DNA samples of 300 participants were genotyped for GC-rs2282679 polymorphism with the ARMS PCR method. PCR primers were designed through Gene Runner software and a specificity check was performed using the BLAST program. The primer sequences designed in this study are listed in Supplementary Table 1. Thermal cycling was carried out using the following program: After initial denaturation at 95°C for 5 min, each cycle with 94°C (30 s), 57°C (35 s), and 72°C (30 s) for 30 cycles. The final extension was at 72°C (5 min) (ABI applied Biosystems 2720 PCR thermal cycler). PCR products were assessed by electrophoresis on a 2% agarose gel.

### Statistical analysis

Continuous variables are presented as mean (SD) for normal and median (IQR) for skewed variables and variables with categorical distribution by numbers and percentages. The Shapiro–Wilk test and histograms were used for testing the normality of data distribution. Differences in participants’ baseline characteristics based on genotype groups in overweight and obese children and adolescents were assessed using the analysis of variance, Kruskal-Wallis H test, and Chi-square test for normal, skewed, and categorical variables, respectively. The genotype frequencies were analyzed using the chi-square test for compliance with Hardy-Weinberg equilibrium (22). VDD was defined as serum levels of 25(OH)D less than 20 ng/mL. Logistic regression analysis was applied to compare the prevalence of VDD between intervention groups, followed by subgroup analysis regarding rs2282679 genotypes [i.e., TT, TG, and GG] after controlling for baseline age, 25(OH)D concentration, and season. The interaction effect of type of genotypes × times × groups of supplementation was evaluated in mixed effects models with fixed and random individual-level effects and random slopes. The interaction effect assessed the differences in 25(OH)D value at different genotypes and dosages over the duration of supplementation. It is noteworthy to mention that we considered random effects in our study in which every participant could have different changes. All statistical analyses were performed in STATA version 14 (STATA, College Station, TX, USA); the significance level was set at *P* < 0.05 (two-tailed).

## Results

### Study population

Table 1 shows baseline characteristics of participants by rs2282679 genotype groups in overweight and obese children and adolescents. The mean ± SD of age and BMI Z-score in all participants were 9.3 ± 1.7 years and 2.58 ± 0.74 kg/m^2^, respectively. The mean of 25(OH)D values was not different between the three genotype groups (*P* = 0.062); the 25(OH)D levels in participants with TT, TG, and GG genotype was 15.4, 14.4, and 10.8 ng/mL, respectively. Children and adolescents with the TT genotype were taller 141.6 ± 10.7 than others (*P* = 0.044). None of the dietary factors, including energy (*P* = 0.512), vitamin D (*P* = 0.106), calcium (*P* = 0.119), magnesium (*P* = 0.841), and phosphorus (*P* = 0.755) intakes were different between the three genotype groups.

**TABLE 1:** Baseline characteristics of participants according to GC-rs2282679 genotypes in overweight and obese children and adolescents.

Characteristics	Overall (*n* = 300)	TT (*n* = 126)	GG (*n* = 30)	TG (*n* = 144)	*P*-Value
Age (years)	9.28 ± 1.72	9.15 ± 1.73	9.17 ± 1.89	9.42 ± 1.66	0.399
Female [*n* (%)]	137 (45.7)	64 (50.8)	11 (36.7)	62 (43.1)	0.258
Weight (kg)	46.6 ± 12.9	44.8 ± 12.6	46.7 ± 17.5	48.1 ± 11.9	0.113
Height (cm)	140.0 ± 11.1	138.2 ± 11.1	139.0 ± 11.7	142.0 ± 10.7	**0.044**
Z-score height	0.90 ± 1.02	0.71 ± 1.08	0.98 ± 1.14	1.05 ± 0.91	**0.023**
Body mass index (kg/m^2^)	23.4 ± 3.4	23.0 ± 3.7	23.7 ± 3.2	23.3 ± 4.8	0.318
Z-score body mass index	2.58 ± 0.74	2.48 ± 0.67	2.61 ± 0.90	2.66 ± 0.75	0.149
Percent of body fat (%)	26.4 ± 7.0	26.6 ± 7.2	24.7 ± 7.0	26.6 ± 6.8	0.351
Soft lean mass (kg)	30.8 ± 7.4	29.7 ± 7.3	31.1 ± 9.6	31.8 ± 6.8	0.075
25-hydroxyvitamin D (ng/mL)	14.4 ± 9.5	15.4 ± 10.6	10.8 ± 4.8	14.4 ± 9.2	0.062
Physical activity (MET/h/week)	9.5 (2.2–26.4)	10.6 (2.2–29.4)	8.9 (3.2–32.0)	9.5 (2.0–24.3)	0.679
**Sun exposure [*n* (%)]**					
< 15 min	109 (36.3)	46 (36.5)	14 (46.7)	49 (34.0)	0.604
15–30 min	115 (38.3)	47 (37.3)	8 (26.7)	60 (41.7)	
30–60 min	76 (25.3)	33 (26.2)	8 (26.7)	35 (24.3)	
**Dietary intake/1,000 Kcal/day**					
Energy (kcal)	1,756 (1,389–2,082)	1,717 (1,359–1,974)	1,844 (1,405–2,173)	1,776 (1,398–2,173)	0.512
Calcium (mg)	360 (253–487)	345 (230–442)	353 (225–491)	378 (272–500)	0.119
Vitamin D (μg)	0.41 (0.00–1.19)	0.31 (0.00–1.18)	0.70 (0.36–1.92)	0.33 (0.00–1.17)	0.106
Magnesium (mg)	104 (84–132)	104 (88–129)	102 (76–134)	104 (82–132)	0.841
Phosphorus (mg)	508 (395–628)	496 (381–615)	501 (392–618)	512 (406–629)	0.755

Data are given as the mean ± SD or median (IQ 25–75) unless otherwise indicated. The values of *P*-value column that were bolded were less than 0.05 which were considered as a significant levels.

No significant differences were found in the mean values of 25(OH)D among three genotypes at the end of the intervention (*P*-value = 0.64); participants with TG genotypes were taller (*P*-value < 0.01), as well as they, had higher values of soft lean mass (*P*-value = 0.02), and weight (*P*-value = 0.02) compared to participants with TT genotype. There was no significant difference between genotype groups in energy, vitamin D, calcium, magnesium, and phosphorus intake (all *P*-values > 0.05, Supplementary Table 2).

### Genotyping

The PCR products were separated by size via agarose gel electrophoresis and analysis of the products showed 281 and 665 bp bands lane for rs2282679 (Supplementary Figure 1). Accuracy of results was confirmed by direct sequencing of 10% samples using outer primers. The rs2282679 SNP T > G was in Hardy-Weinberg equilibrium, X^2^ testing; *P*-value > 0.05. The minor allele frequency of rs2282679 (G allele as a minor allele) in our study was 0.34.

### Effect of vitamin D supplementation and rs2282679 genotypes on the vitamin D status

A significant reduction in VDD was observed after 6 and 12 months of vitamin D supplementation with dosages of 1,000 or 2,000 IU compared to 600 IU; adjusted odds ratios ranged from 0.13 to 0.37; all *P*-values were <0.05 (Table 2).

**TABLE 2:** Odds ratios (95% confidence interval) of vitamin D deficiency among intervention groups.

	600 IU	1,000 IU	2,000 IU
After 6 months of supplementation	1.00	0.31 (0.14–0.66)	0.26 (0.12–0.56)
After 12 months of supplementation	1.00	0.37 (0.17–0.81)	0.13 (0.05–0.37)

IU, international unit. The data were adjusted for age, baseline 25(OH)D concentration, and season.

**FIGURE 1:**
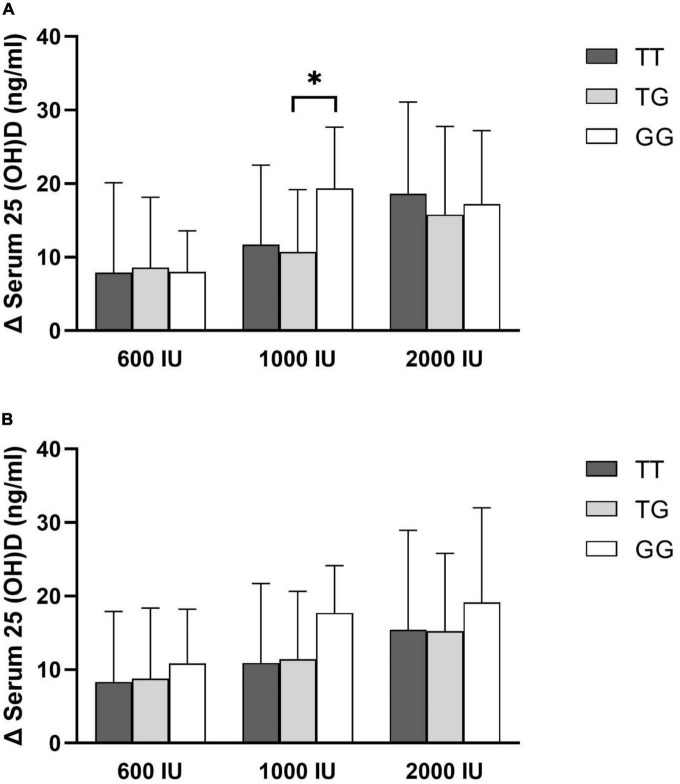
Changes in serum 25(OH)D concentration by rs2282679 polymorphism in each treatment group. **(A)** 6-month change, **(B)** 12-month change.

The mean changes in serum 25(OH)D levels after 6 or 12 months of supplementation in each intervention group did not differ significantly among the genotypes, except individuals with the GG genotype showed more remarkable incremental changes in serum 25(OH)D levels in the 1,000 IU vitamin D group after 6 months of supplementation than those with the TG genotype (*P* value = 0.043, Figure 1).

**FIGURE 2:**
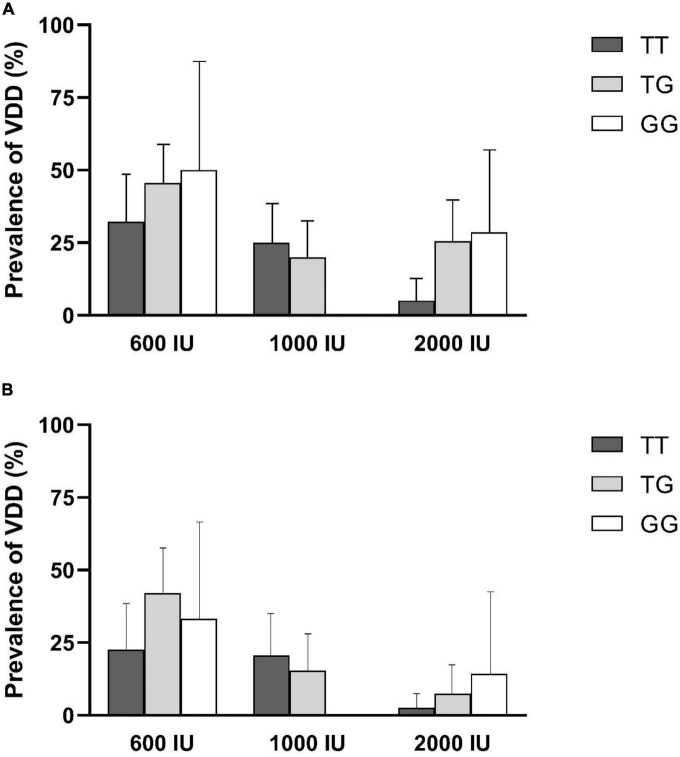
Prevalence of vitamin D deficiency (VDD) by rs2282679 polymorphism in each treatment group. **(A)** 6-month percentage of VDD, **(B)** 12-month percentage of VDD.

In addition, the prevalence of VDD (95% confidence interval), after 6- or 12-month supplementation was shown in Figure 2. The lowest prevalence of VDD in 600 IU or 2,000 IU of intervention groups was observed in individuals with TT genotypes after 6 or 12 months of supplementation. In the intervention group receiving 1,000 IU of vitamin D, the prevalence of VDD after 6 or 12 months was higher in individuals with the TT genotype than those with the TG genotype, and for individuals with the GG genotype, VDD was no longer prevalent following 6 or 12 months of intervention.

No significant association of genotypes with risk of VDD was observed in each intervention arms at 6- or 12 months of supplementation, except, that individuals in 2,000 IU group with TG genotype showed a higher risk of VDD compared to those with TT genotype after 6 months of supplementation [odds ratio (95% CI): 6.94; 1.30–37.02, Table 3].

**TABLE 3:** Odds ratios (95% confidence interval) of vitamin D deficiency among GC-rs2282679 genotypes stratified by intervention groups.

	TT	TG	GG
**After 6 months of supplementation**			
600 IU (*n* = 83)	1.00	1.27 (0.44–3.71)	1.32 (0.22–7.75)
1,000 IU (*n* = 85)	1.00	0.84 (0.24–2.87)	–
2,000 IU (*n* = 89)	1.00	6.94 (1.30–37.02)	7.24 (0.74–70.91)
**After 12 months of supplementation**			
600 IU (*n* = 75)	1.00	2.15 (0.70–6.54)	1.36 (0.19–9.38)
1,000 IU (*n* = 82)	1.00	0.78 (0.21–2.85)	–
2,000 IU (*n* = 85)	1.00	3.10 (0.29–32.96)	4.26 (0.22–83.05)

IU, international unit. The data were adjusted for age, baseline 25(OH)D concentration, and season.

Table 4 indicates the interactions of clinical measurements based on the type of genotypes, times, and groups of supplementation in overweight and obese children and adolescents. No significant interaction was found between the TT genotype during 6 or 12 months of 1,000 or 2,000 IU supplementation with the serum level of 25(OH)D. In other words, there was no synergistic effect on 25(OH)D value through TT genotype, dosage, and the duration of supplementation. Similarly, the same results were observed regarding two other types of genotype, dosage, and time duration with 25(OH)D concentration. In addition, we observed no interaction between time duration, three types of genotype, and 1,000 and 2,000 IU vitamin D supplements with calcium, phosphorus, and PTH levels.

**TABLE 4:** Interactions of clinical measurements based on type of genotypes, time of measurement, and group of intervention in overweight and obese children and adolescents.

	β (95% CI)	*P*-Value
**25(OH)Vitamin D (ng/mL) (genotype × time × group)**		
TT **×** 6 months **×** 1,000 IU	−8.30 (−18.28, 1.66)	0.103
TT **×** 12 months **×** 1,000 IU	−5.40 (−15.86, 5.06)	0.312
TG **×** 6 months **×** 1,000 IU	−9.34 (−19.10, 0.41)	0.061
TG **×** 12 months **×** 1,000 IU	−4.66 (−14.94, 5.62)	0.374
TT **×** 6 months **×** 2,000 IU	0.74 (−9.90, 11.38)	0.891
TT **×** 12 months **×** 2,000 IU	−2.48 (−13.58, 8.60)	0.660
TG **×** 6 months **×** 2,000 IU	−1.66 (−12.12, 8.78)	0.755
TG **×** 12 months **×** 2,000 IU	−1.70 (−12.66, 9.25)	0.761
**Calcium (mg/dL) (genotype × time × group)**		
TT **×** 6 months **×** 1,000 IU	−0.22 (−1.10, 0.64)	0.611
TT **×** 12 months **×** 1,000 IU	−0.01 (−0.92, 0.89)	0.975
TG **×** 6 months **×** 1,000 IU	−0.83 (−1.68, 0.02)	0.057
TG **×** 12 months **×** 1,000 IU	−0.50 (−1.39, 0.39)	0.273
TT **×** 6 months **×** 2,000 IU	0.35 (−0.60, 1.31)	0.470
TT **×** 12 months **×** 2,000 IU	0.27 (−0.72, 1.26)	0.593
TG **×** 6 months **×** 2,000 IU	0.23 (−0.70, 1.18)	0.619
TG **×** 12 months **×** 2,000 IU	0.19 (−0.78, 1.18)	0.692
**Phosphorus (mg/dL) (genotype × time × group)**		
TT **×** 6 months **×** 1,000 IU	−0.54 (−1.39, 0.29)	0.204
TT **×** 12 months **×** 1,000 IU	−0.43 (−1.31, 0.44)	0.332
TG **×** 6 months **×** 1,000 IU	−0.64 (−1.47, 0.17)	0.125
TG **×** 12 months **×** 1,000 IU	−0.68 (−1.55, 0.17)	0.120
TT **×** 6 months **×** 2,000 IU	−0.46 (−1.41, 0.49)	0.342
TT **×** 12 months **×** 2,000 IU	−0.33 (−1.32, 0.65)	0.504
TG **×** 6 months **×** 2,000 IU	−0.71 (−1.65, 0.22)	0.135
TG **×** 12 months **×** 2,000 IU	−0.69 (−1.67, 0.27)	0.162
**Alkaline phosphatase (IU/L) (genotype × time × group)**		
TT **×** 6 months **×** 1,000 IU	−127.38 (298.37, 43.60)	0.144
TT **×** 12 months **×** 1,000 IU	−137.95 (−317.84, 41.93)	0.133
TG **×** 6 months **×** 1,000 IU	−70.72 (−238.10, 96.65)	0.408
TG **×** 12 months **×** 1,000 IU	−55.76 (−232.75, 121.22)	0.537
TT **×** 6 months **×** 2,000 IU	−67.00 (−247.86, 113.85)	0.468
TT **×** 12 months **×** 2,000 IU	−82.12 (−271.23, 106.98)	0.395
TG **×** 6 months **×** 2,000 IU	−96.92 (−274.46, 80.62)	0.285
TG **×** 12 months **×** 2,000 IU	−101.08 (−287.93, 85.75)	0.289
**Parathyroid hormone (pg/mL) (genotype × time × group)**		
TT **×** 6 months **×** 1,000 IU	−20.60 (−58.77, 17.56)	0.290
TT **×** 12 months **×** 1,000 IU	−17.10 (−57.12, 22.90)	0.402
TG **×** 6 months **×** 1,000 IU	−1.62 (−38.97, 35.71)	0.932
TG **×** 12 months **×** 1,000 IU	3.80 (−35.53, 43.14)	0.850
TT **×** 6 months **×** 2,000 IU	−14.26 (−55.03, 26.50)	0.493
TT **×** 12 months **×** 2,000 IU	−22.45 (−64.91, 20.01)	0.300
TG **×** 6 months **×** 2,000 IU	22.01 (−18.01, 62.05)	0.281
TG **×** 12 months **×** 2,000 IU	−4.66 (−46.60, 37.27)	0.827

## Discussion

Previously, we found that 1,000 and 2,000 IU vitamin D supplementation could increase our participants’ 25(OH)D concentration (16). In the present study, we investigated whether VDD can be treated by vitamin D supplementation with considering rs2282679 genotypes. No interaction was found between different intervention doses with TT, TG, and GG genotypes, and 6- or 12-month 25(OH)D measurements. Moreover, other factors, including the serum levels of calcium, phosphorus, PTH, and ALP, did not alter with these interactions during 1-year supplementation of vitamin D.

After 6 months of supplementation with a dose of 1,000 IU of vitamin D, the changes in the serum level of vitamin D in individuals with the GG genotype were significantly higher than in those with the TG genotype. Due to the small sample size in the GG group (*n* = 9) compared to those in the TG group (*n* = 40), this significant difference should be interpreted with caution. Additionally, the prevalence of VDD disappeared in individuals with the GG genotype after supplementation with 1,000 IU of vitamin D.

Twins and family studies indicated a substantial genetic basis for variability in 25(OH)D, with estimates of heritability up to 86% (23, 24). The majority of circulating 25(OH)D is bound to DBP (85–90%), and albumin (10–15%), with less than 1% free (7). In light of its biological significance, several studies (9, 25–28) demonstrated that GC polymorphism account for variation in total circulating 25(OH)D concentrations as well as the association of genetic variants of DBP and the response to vitamin D supplementation (13–15, 29–33). In contrast to our findings, individuals who carry the minor allele for rs22282679 appear to have lower baseline vitamin D levels and are less likely to respond to vitamin D supplementation (13–15, 31, 32). It should be mentioned that slow et al. showed that GC common variants (especially rs2282679) were correlated with 25(OH)D concentrations following supplementation. However, this effect was relatively small and disappeared after >2 months of supplementation (14). It could be assumed that some variants of GC could be a saturable factor affecting the response to supplementation, which would be dose- and time-dependent. As a result, it appears that if vitamin D supplementation is administered for a sufficient period of time, even supplementation with routine doses of vitamin D can overcome the genetic differences in GC. Alternatively, considering the highly polymorphic nature of the GC gene, it may be also possible that the differences in individual responses to vitamin D supplementation may be a consequence of other SNPs which have not been investigated. Interestingly, Mehramiz et al. have shown that GC-rs4588 polymorphism appears to modulate the healthy adolescent Iranian girls response to supplementation with a dosage of 50,000 IU of vitamin D_3_ for 9 weeks (34).

It has been suggested that other genetic variants involved in vitamin D metabolism, such as CYP2R1 (Vitamin D 25-hydroxylase) (28), VDR (vitamin D receptor) (35), and RXRA (Retinoid X Receptor Alpha) (33), also play an important role in response to vitamin D supplementation. In addition, Khayyatzadeh et al. have demonstrated that the response of adolescent Iranian girls with a mean age of 15 years to 9 weeks supplementation with a dosage of 50,000 IU of vitamin D_3_ weekly, modulated by the rs10741657 variant of the CYP2R1 gene (36).

In addition to genetic predisposition, a study of 2,187 healthy adults found that the response to cholecalciferol supplementation was affected by lower baseline serum 25(OH)D levels, seasonal changes, and UVB sunlight (37). Another mechanism that can link to people’s response to vitamin D supplementation is obesity. It was demonstrated that obese individuals had hypovitaminosis D which is because of the low bioavailability of vitamin D and its deposition in body fat (38). In this regard, obesity may nullify the association between genetic predisposition and response to vitamin D supplementation.

Vitamin D genetic variations are extensive and complex according to their functions. Similar to the polymorphism genotyped in our study, Santos et al. revealed that the rs2282679 polymorphism of DBP was associated with susceptibility to VDD in Brazilian women (27). In Japanese children, it was manifested that VDR, NADSYN1 (NAD Synthetase 1), and GC polymorphisms can be linked to VDD (26). It has been shown that CYP2R1 plays a role in encoding the critical enzyme that converts vitamin D to 25(OH)D in the liver (39). In this way, it may affect 25(OH)D synthesis as well as serum concentration (9, 25, 40). Tomei et al. indicated that vitamin D status could be related to both rs731236 (VDR gene) and rs7116978 (CYP2R1 gene) in women (40). Furthermore, another research conducted on children 8–11 years old found that common genetic variants such as rs10500804 (CYP2R1 gene), rs4588, and rs7041 (GC gene) had associations with lower 25(OH)D values which could be related to the different seasons and UVB sunlight exposure (25).

Our study had some strengths that needed to be pointed out. We did a randomized clinical trial study that lasted 12 months, which is long enough to see changes in the outcome of interest. To the best of our knowledge, it was the first research to investigate the effect of vitamin D supplementation on the risk of VDD with the interaction of genotype variants in overweight and obese children and adolescents. Also, we considered the interaction of the duration of intervention and 1,000 and 2,000 IU vitamin D dosages with rs2282679 polymorphism. It also had some limitations. In this study, only one polymorphism associated with DBP was genotyped. There might be possible interactions between polymorphisms of other genes involved in vitamin D metabolism with response to vitamin D supplementation. Furthermore, VDBP, free 25(OH)D, and other vitamin D metabolites were not measured in the blood or urine. It has been proposed to measure the free fraction of 25(OH)D as well as the ratio of 24,25-dihydroxy vitamin D (24,25 [OH]2D) to 25(OH)D.

In light of recent studies that have investigated the individual’s response to vitamin D supplementation by considering a variety of SNPs associated with the vitamin D metabolic pathway, it has been recommended to develop some Genetic risk scores (GRS) based on the number of known vitamin D-related SNPs.

## Conclusion

Time, dosages, and rs2282679 polymorphism of DBP did not an interaction with the effect of vitamin D supplementation on VDD in overweight and obese children and adolescents. It seems that supplementation with doses of 1,000 and 2,000 IU of vitamin D significantly reduced the risk of VDD compared to 600 IU without considering rs2282679 genotypes; in addition, no superiority of 2,000 IU compared to 1,000 IU response to vitamin D supplementation was seen. Further study is needed to investigate more polymorphisms in the metabolic pathway of vitamin D, especially in children and adolescents at risk of vitamin D deficiency.

## Data availability statement

The datasets used and/or analyzed during the current study are available upon reasonable request through the corresponding authors PM, mirmiran@endocrine.ac.ir; parvin.mirmiran@gmail.com and FH, fhospanah@ebdocrine.ac.ir.

## Ethics statement

The studies involving human participants were reviewed and approved by the Ethics Research Council of the Research Institute for Endocrine Sciences, Shahid Beheshti University of Medical Sciences approved the protocol of this study. Written informed consent to participate in this study was provided by the participants’ legal guardian/next of kin.

## Author contributions

GA and EY conceptualized and designed the study, interpreted the data, and prepared the manuscript. AN and MM analyzed the data and drafted the initial manuscript. LN and MD extracted the DNA and genotyped and drafted the initial manuscript. FH and PM supervised the project and drafted the initial manuscript. All authors contributed to the article and approved the submitted version.
