# Supplementary figures and images for: Impact of daily vitamin D3 supplementation on the risk of vitamin D deficiency with the interaction of rs2282679 in vitamin D binding protein gene (GC) among overweight and obese children and adolescents: A one-year randomized controlled trial
Source: Front Nutr. 2022 Dec 12;9:1061496. doi: 10.3389/fnut.2022.1061496 (PMC9792175; doi:10.3389/fnut.2022.1061496)

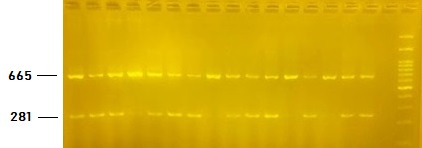

Supplement: Supplementary file 1 [file Image_1.JPEG]
