# Supplementary material for: Impact of daily vitamin D3 supplementation on the risk of vitamin D deficiency with the interaction of rs2282679 in vitamin D binding protein gene (GC) among overweight and obese children and adolescents: A one-year randomized controlled trial
Source: Front Nutr. 2022 Dec 12;9:1061496. doi: 10.3389/fnut.2022.1061496 (PMC9792175; doi:10.3389/fnut.2022.1061496)
Supplement: Supplementary file 2 [file Table_1.DOCX]

**Supplementary Table1-** Polymerase chain reaction primers sequence.

| SNP | A sequence of primer (5′-3′ end) | TM (C˚) |
| --- | --- | --- |
| rs2282679 (GC) | Forward wild type (T): CAAATCTCTGTCTCTTAATTATCTCAtAA | 56 |
|  | Forward mutant (G): CAAATCTCTGTCTCTTAATTATCTCAtAC | 56 |
|  | Common reverse: GCTGCTATGGTTCTACAGTTCCA | 56 |
|  | Forward Seq Primer: TGTTCAAGTCACACTCAGCCTC | 57 |
