# Supplementary material for: Impact of daily vitamin D3 supplementation on the risk of vitamin D deficiency with the interaction of rs2282679 in vitamin D binding protein gene (GC) among overweight and obese children and adolescents: A one-year randomized controlled trial
Source: Front Nutr. 2022 Dec 12;9:1061496. doi: 10.3389/fnut.2022.1061496 (PMC9792175; doi:10.3389/fnut.2022.1061496)
Supplement: Supplementary file 3 [file Table_2.DOCX]

**Supplementary Table 2-** Characteristics of participants by GC-rs2282679 genotype groups at the end of the intervention.

| **Characteristics** | **Overall** | **TT** | **GG** | **TG** | **p-value** |
| --- | --- | --- | --- | --- | --- |
| Age (years) | 10.5 ± 1.7 | 10.4 ± 1.6 | 10.3 ± 2.0 | 10.7 ± 1.6 | 0.25 |
| Female, n (%) | 137 (45.7) | 64 (50.8) | 11 (36.7) | 62 (43.1) | 0.26 |
| Weight (kg) | 54.4 ± 12.9 | 52.1 ± 11.9 | 51.5 ± 14.5 | 56.9 ± 13.2 | **0.01** |
| Height (cm) | 147.1 ±11.1 | 144.9 ± 10.9 | 146.2 ± 11.6 | 149.3 ± 10.9 | **0.01** |
| Z-score height | 0.90 ±0.98 | 0.7 ± 1.0 | 0.9 ± 1.1 | 1.1 ± 0.9 | **0.02** |
| Body mass index (Kg/m^2^) | 24.8 ± 3.3 | 24.5 ± 3.1 | 23.7 ± 3.5 | 25.2 ± 3.4 | 0.08 |
| Z-score body mass index | 2.53 ± 0.66 | 2.5 ± 0.6 | 2.4 ±0.7 | 2.6 ± 0.7 | 0.54 |
| Percent of body fat (%) | 27.0 ± 5.3 | 26.9 ± 5.3 | 24.6 ± 6.2 | 27.5 ± 5.0 | 0.07 |
| Soft lean mass (kg) | 36.2 ±7.4 | 34.9 ± 7.1 | 35.4 ± 8.5 | 37.6 ± 7.2 | **0.03** |
| 25-hydroxyvitamin D (ng/ml) | 26.8 ± 7.8 | 27.4 ± 7.8 | 27.0 ± 8.7 | 26.3 ± 7.6 | 0.64 |
| Physical activity (MET/h/week) | 3.5 (0.0-12.5) | 4.6 (0.0-13.9) | 1.5 (0.0-10.7) | 3.3 (0.0-10.7) | 0.41 |
| Dietary intake/1000 Kcal/day |  |  |  |  |  |
| Energy (kcal)  Calcium (mg)  Vitamin D (µg)  Magnesium (mg)  Phosphorus (mg) | 1778 (1500-2097)  314 (240-422)  0.26 (0.00 - 0.83)  101 (83-118)  461 (382-555) | 1842 (1523-2108)  293 (240-400)  0.35 (0.00-0.80)  98 (82-116)  456 (383-542) | 1863 (1607-2119)  274 (220-536)  0.26 (0.00-1.18)  102 (82-122)  365 (473-597) | 1675 (1471-2088)  338 (241-430)  0.25 (0.01-0.88)  101 (83-119)  468 (378-556) | 0.81  0.43  0.16  0.83  0.97 |

Data are given as the mean ± SD or median (IQ 25–75) unless otherwise indicated.
